# Supplementary material for: Combining rapid diagnostic tests to estimate primary and post-primary dengue immune status at the point of care
Source: PLoS Negl Trop Dis. 2022 May 4;16(5):e0010365. doi: 10.1371/journal.pntd.0010365 (PMC9067681; doi:10.1371/journal.pntd.0010365)
Supplement: S1 File — (DOCX) [file pntd.0010365.s004.docx]

# **Methods used to determine reference primary and post-primary dengue immune status**

The primary and post-primary immune status of the sample population categorised according to a previous developed algorithm ^1^. Suspected dengue patients either PCR positive or with IgM panbio units≥9.9 were classified as active dengue infections, while patients PCR negative and with IgM panbio units<9.9 were considered non-active dengue infections. Among active dengue infections, those on disease day 1 or 2 with IgG panbio units above and below 2.2 panbio units were categorised as post-primary and primary, respectively. Active cases on disease 3-5, with IgG:IgM ratios above and below 0.45 were classified as post-primary and primary, respectively. Non-active dengue infections were further classified as historical or negative for dengue if they had IgG panbio units above and below 2.2 panbio units, respectively. Post-primary dengue infections include infections with at least one previous flaviviral infection:


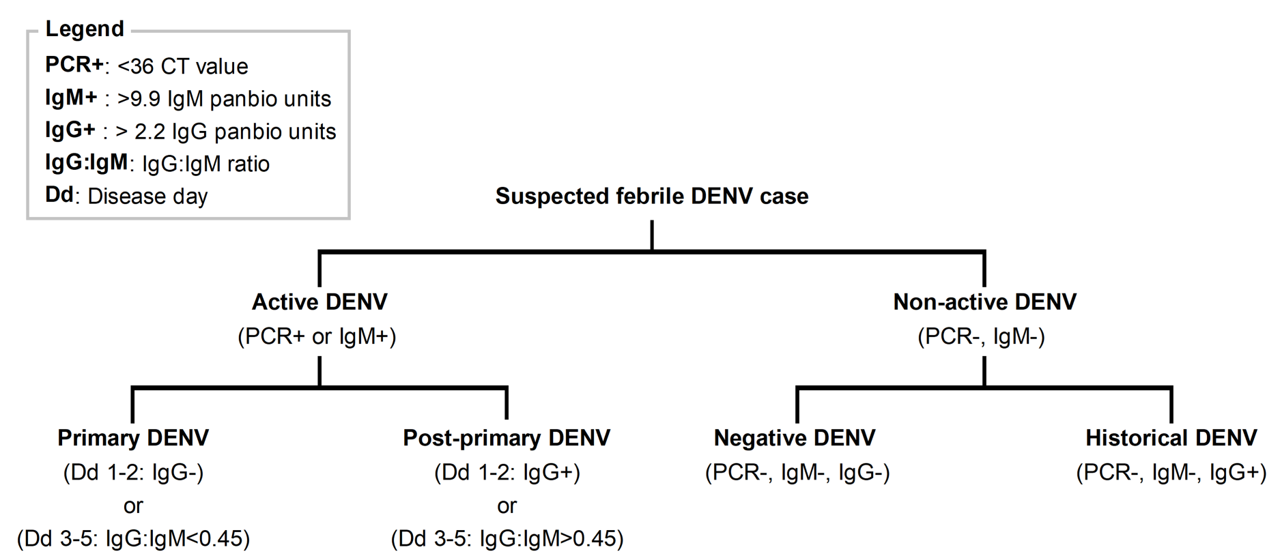


Reference

1. Biggs JR, Sy AK, Brady OJ, *et al*. A serological framework to investigate acute primary and post-primary dengue cases reporting across the Philippines. *BMC Med*. 2020; 18: 364.
