# Supplementary figures and images for: Combining rapid diagnostic tests to estimate primary and post-primary dengue immune status at the point of care
Source: PLoS Negl Trop Dis. 2022 May 4;16(5):e0010365. doi: 10.1371/journal.pntd.0010365 (PMC9067681; doi:10.1371/journal.pntd.0010365)

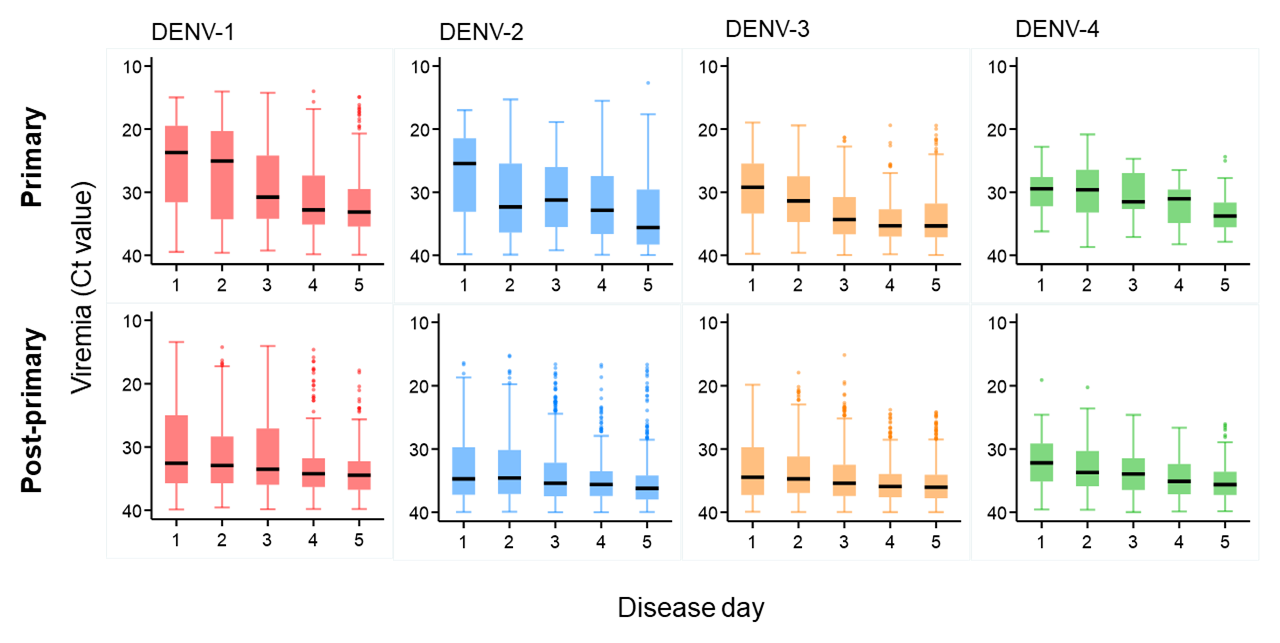

Supplement: S1 Fig — (TIF) [file pntd.0010365.s005.tif]

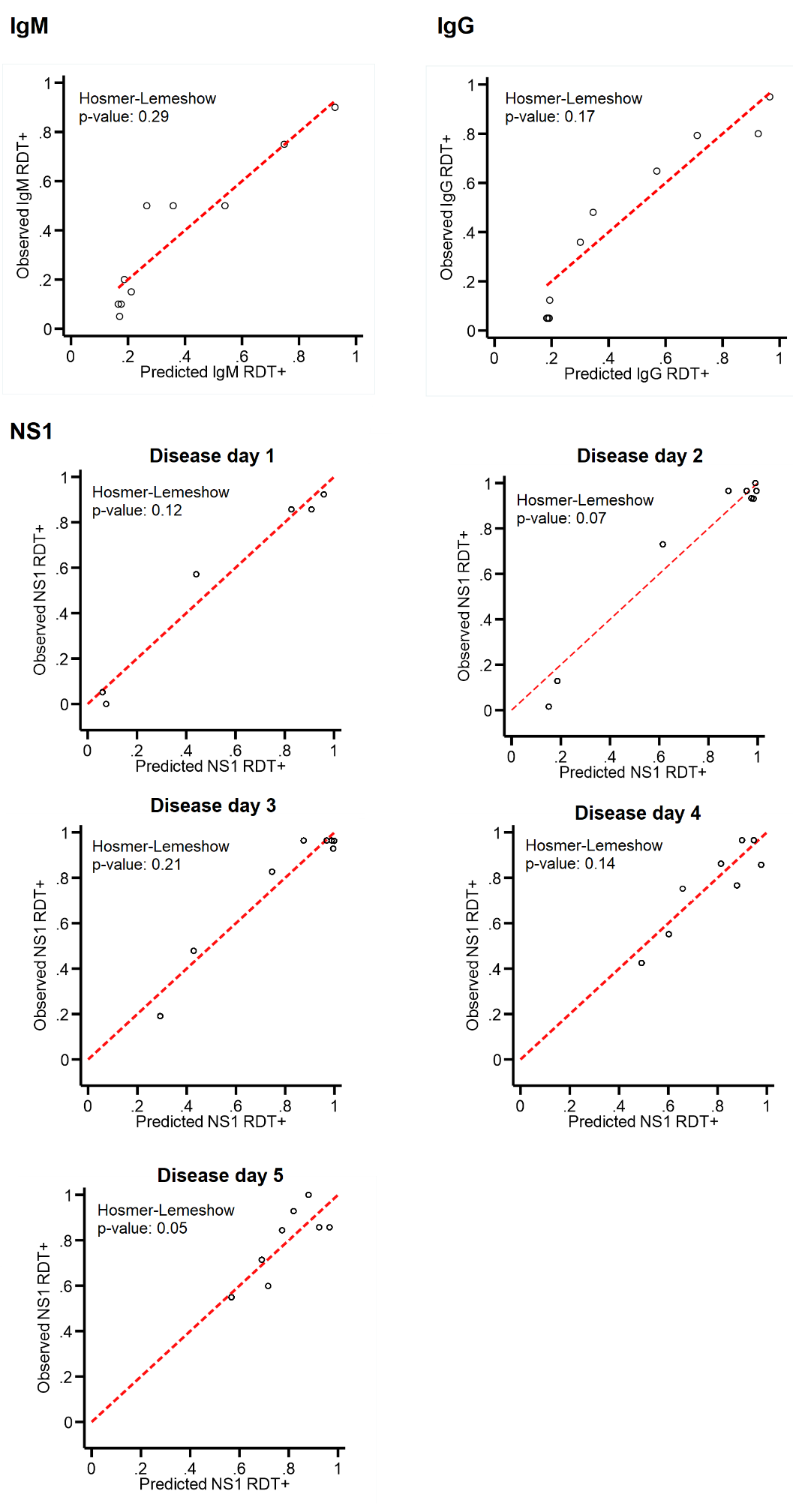

Supplement: S2 Fig — p-values >0.05 infer good model fit. (DOCX) [file pntd.0010365.s006.docx]
