# Supplementary material for: Combining rapid diagnostic tests to estimate primary and post-primary dengue immune status at the point of care
Source: PLoS Negl Trop Dis. 2022 May 4;16(5):e0010365. doi: 10.1371/journal.pntd.0010365 (PMC9067681; doi:10.1371/journal.pntd.0010365)
Supplement: Attachment — Submitted filename: Author_response_to_reviews.docx [file pntd.0010365.s007.docx]

**Author’s response to reviews**

Dear Editor and reviewers,

Re: Combining rapid diagnostic tests to estimate primary and post-primary dengue immune status at the point of care. PLOS NTD (PNTD-D-21-01651)

We are submitting a revised version of the manuscript titled “Combining rapid diagnostic tests to estimate primary and post-primary dengue immune status at the point of care” for publication in PLOS NTD. We thank the editor and reviewers for their insightful comments and useful suggestions. We have responded to each comment below and made changes to the updated manuscript. We hope this clarifies issues raised and look forward to hearing from you. Please note, the references (see line x-x) below that refer to where the changes have been made refer to the clean revised manuscript NOT the revised manuscript with tracked changes.

Sincerely yours,

Joseph Biggs, MSc

Research Assistant

Department of Infection Biology

London School of Hygiene and Tropical Medicine

Email: joseph.biggs1@lshtm.ac.uk

**Editor**

The reviewers expressed interests in a discussion or comparison of the method proposed in this study with other protocols published that have been used to classify primary and post-primary dengue infections, and how Zika infection would interfere with the analyses

**Response:** We agree with the editor about the importance of Zika transmission on our results. We have made substantial revisions in our manuscript that discuss the implications of ZIKV on our results (see responses below to reviewer 1#). The editor is also correct to highlight there are alternative methods for categorising DENV immune status using 1 single serum sample. According to the laboratory tests we used, there are two approaches to determine primary and post-primary immune status. One includes a novel algorithm and another is a commercial (Panbio®) method [1]. Prior to this study we evaluated both these approaches and compared them to the WHO gold standard using paired sera from a separate dataset. The novel algorithm achieved excellent serological agreement with gold standard (90.5%) while the panbio method achieved 71.4% agreement. Moreover, the panbio method was able to classify only a much smaller proportion of patients (30% less) than the novel algorithm. It was therefore concluded the commercial method for categorising immune status was inferior and deemed inadequate to classify immune status. Therefore, in this study we refrained from categorising the immune status using the panbio method as we are confident the elicited results would be misleading. This is discussed in more detail in the introduction section of this manuscript: *“… Prior to this study, we generated and validated an algorithm capable of distinguishing individual primary and post-primary DENV immune status that relies on basic epidemiological and laboratory-obtained metrics from single serum samples [12]. The framework utilises individual molecular and serological DENV metrics derived from the CDC fourplex DENV1-4 PCR assay and commercial IgM and IgG capture ELISAs (Panbio®, Abbott, Cat no. 01PE10 & 01PE20), respectively. Panbio® serological assays were chosen based on a WHO report which compared their performance to other commercial assays which revealed similar sensitivities [31]. The novel algorithm achieved 90% agreement with the WHO gold standard method for categorising immune status based on paired sera and proved superior to the Panbio® method for classifying immune status [11,12]…”* (see section: Introduction, page 9, lines: 269-278).

**Reviewer 1#**

***Methods***

Reviewer #1: The study presented by Biggs et al. is well-conducted and included appropriate methods and analysis. However, this reviewers finds that there are some concerns to be addressed to improve the clarity and interpretability of their work.

1.a. Regarding the methodology, and although the study has been properly conducted, the first limitation that came to my attention was the choice of the standard serologic assay. Was any kind of accuracy comparison performed among the panbio test and other commercially available kits that would justify the choice of the first?

**Response:** The standard serologically assays were selected prior to this study yet were based on a WHO report which compared their performance to other commercial brands [2,3]. Overall Panbio® assays have good sensitivity levels compared to other commercial brands. Panbio assays were therefore used in a study prior to this which explained how the immune status algorithm was generated and validated [1]. In the introduction we state more clearly the justifications for using pabio kits: *“…Prior to this study, we generated and validated a single serum sample algorithm capable of distinguishing individual primary and post-primary immune status that relies on basic epidemiological and laboratory-obtained metrics [12]. The framework utilises individual molecular and serological DENV metrics derived from the CDC fourplex DENV1-4 PCR assay and commercial IgM and IgG capture ELISAs (Panbio®, Abbott, Cat no. 01PE10 & 01PE20), respectively. Panbio® serological assays were chosen based on a WHO report which compared their performance to other commercial assays [31]. The algorithm achieved 90% agreement with the WHO gold standard method for categorising immune status based on paired sera and proved superior to the Panbio® method for classifying immune status [11,12] …).* (See section: Introduction, page 9, lines: 248-257).

1.b. The authors state on page 11, lines 321-323 that participants’ DENV immune status classification was performed based on methods described in the reference #12. Although, unnecessary repetition of published data/methods shall be avoided, it is not clear to the reader how this classification was performed. Ref #12 describes the development of an algorithm to properly differentiate active from previous DENV infections based on demographic and serologic data and comparisons to WHO gold standard. In the current manuscript, it is not clear if the authors kept the same rationale or simply used the cut-off suggested by the Panbio test manufacturer.

**Response:** We agree with reviewer 1# that the reference immune status classification the study population, based on the previously developed algorithm and laboratory methods, needs to be made clearer in this manuscript. In the methods section it now reads: “…*Using laboratory and basic epidemiological data, we categorized the reference DENV immune status (primary, post-primary, historical and negative) of the entire study population using the exact methods described in [12]. Patients who reported as either PCR+ or IgM+ (Ct≤36 or IgM panbio units>9.9) were classified as active DENV infections as both these markers are detectable during infection. PCR- and IgM- (IgM panbio units<9.9) cases were categorised as non-active DENV infections and represent patients misdiagnosed as suspected active dengue. Non-active DENV infections were further classified as historical or negative if they were DENV IgG positive (IgG panbio units>2.2) or negative (IgG panbio units<2.2), respectively. Historical and negative cases included misdiagnosed patients who reported without a current dengue infection with and without previous exposure to dengue, respectively. IgG:IgM ratios (IgG panbio units/IgM panbio units) were used to distinguish active DENV infections as primary or post-primary cases. Among active dengue patients at the early stage of disease (disease day 1 or 2), those DENV IgG+ and IgG- were classed as post-primary and primary respectively. Among active cases on disease day 3 to 5, individuals with IgG:IgM ratios above and below 0.45 were categorised as post-primary and primary respectively. As a consequence of previous findings [12,34], post-primary cases included current DENV infections with at least one previous flavivirus infection including DENV and or ZIKV...”* (see section: Methods, page 12, lines: 345-362)

In addition, we have included the previously generated algorithm as a supplementary figure so readers can better understand how we categorized immune status based on novel methods and not using Panbio thresholds which have previously been shown to be inferior for categorising primary/post-primary DENV immune status [1]. (See S1 file).

***Results***

The conclusions presented by the current manuscript are in agreement with the generated data and performed analysis. However, information regarding cross-reactivity with other flavivirus is not discussed in the results/discussion section, although it is of utmost importance for the present work.

1.c. DENV active infection (as determined by PCR) and DENV serology (as determined by ELISA) seem to be well characterised in the study, however the authors have not included any data regarding cross-reactivity with other co-circulating flaviviruses. Indeed, the authors state on page 11, lines 335-336 that “Post-primary cases include current DENV infections with at least one previous flavivirus exposure.” As cases of flaviviruses co-infection and several hypothesis indicating more severe Dengue or Zika clinical phenotypes associated to previous flavivirus exposure, how would the authors explain the applicability of the proposed diagnostic framework? Also, how did the authors rule out the possibility of flavivirus co-infection and further DENV misclassification?

Response: We agree with reviewer 1#, the impact of ZIKV co-circulation on our results is very important and have now included more detail throughout the manuscript explaining the impact ZIKV has on our generated algorithm for determining DENV immune status.

In the introduction we now discuss our previous findings which revealed prior ZIKV exposure (which was shown to be widespread across the Philippines [4]) primed individuals for a postprimary-like infection instead of a primary-like infection upon their first infection with DENV. The high level of ZIKV IgG found among a proportion of post-primary DENV infections suggested they could have been preceded by either a DENV or a ZIKV infection. As a consequence, reporting post-primary DENV infections are interpreted as a current DENV infection (As they are PCR+/IgM+ for DENV) with prior exposure to flaviviruses as they had elevated DENV IgG and some had elevated ZIKV IgG too. Therefore using such cross-reactive tests we are unable to rule out whether post-primary DENV infections had a prior DENV or ZIKV infection. We also discuss evidence that prior exposure to ZIKV is associated with a more severe subsequent DENV infection. In the introduction it now reads: *“…Given dengue serological assays have been shown to detect cross-reactive antibodies elicited from other flavivirus infections, including Zika virus (ZIKV) which is often co-endemic with dengue [32–34], previous analysis explored the impact of ZIKV exposure on the generated DENV immune status algorithm [12]. Results revealed a proportion of those with post-primary, opposed to primary, DENV infections had evidence of ZIKV IgG but not IgM exposure. This suggested some patients had historical ZIKV exposure that primed individuals for a subsequent post-primary-like, instead of a primary-like, DENV infection upon their first infection with DENV. Moreover, further analysis suggested post-primary DENV infections with prior ZIKV exposure were at risk of adverse clinical symptoms [34] which has been previously reported in Nicaragua [35]. These findings suggested that individuals categorised as post-primary DENV infections include those with either prior exposure to DENV and/or other flaviviruses [12]…”* (See section Introduction, page 9, lines 257-269).

In the methods we now again mention the impact prior ZIKV has on the interpretation of the immune status algorithm: *“…As a consequence of previous findings [12,34], post-primary cases included current DENV infections with at least one previous flavivirus infection including DENV and or ZIKV…”* (See section methods, page 12, line 360-362).

In the discussion section, we now discuss the impact potential widespread co-circulation of ZIKV may have on the translatability between DENV antibody RDTs and ELISAs: *“…In contrast, some with elevated ELISA antibody response were negative for corresponding antibody RDTs. This trend could be attributed to ELISAs, yet not RDTs, cross-reacting with other flaviviruses including ZIKV which has shown to be potentially widespread across dengue-endemic countries [32–34]. It is now well established commercial DENV ELISAs cross-react with ZIKV [42,43] yet whether DENV antibody rapid tests cross-react with ZIKV remains poorly characterised and deserves further attention [44,45]…”* (See Section: Discussion, page 20-21, lines 573-579).

Reviewer 1# also correctly highlights the risk of DENV and flavivirus co-infection on our results. We previously investigated this prior to this study in [1] and found no evidence those reporting with DENV were in fact ZIKV infections. Among those reporting with either primary or post-primary infections, all those sampled had no evidence had elevated ZIKV IgM (although a proportion had elevated ZIKV IgG). ZIKV IgM is a short-term serological marker of recent ZIKV exposure, therefore we concluded the reporting population included only current DENV infections. The introduction now reads: *“…Given dengue serological assays have been shown to detect cross-reactive antibodies elicited from other flavivirus infections, including Zika virus (ZIKV) which is often co-endemic with dengue [32–34], previous analysis explored the impact of ZIKV exposure on the generated DENV immune status algorithm [12]. Results revealed a proportion of those with post-primary, opposed to primary, DENV infections had evidence of ZIKV IgG but not IgM exposure. This suggested some patients had historical ZIKV exposure that primed individuals for a subsequent post-primary-like, instead of a primary-like, DENV infection upon their first infection with DENV”* (See section Introduction, page: 9, lines: 257-264)

***Conclusions***

The conclusions were appropriately drawn based on the authors' findings.

***Data presentation***

A few minor concerns are listed below to improve clarity:

2.a. Dengue testing:
- S1 Table should be replaced by S2 Table on page 11, line 318. Also, please clarify the meaning of each symbol used in the table, according to the main text.

**Response:** In the dengue testing section of the methods, S1 Table is the correct supplementary figure to refer to. In the main text we have made it clearer what this table refers to. *“…A summary of the dengue testing conducted among all three datasets are shown in (S1 Table)…”* (See section: Methods, page: 11, lines 341). We have also clarified the meaning of the symbols in the table to make it clear to the reader the ‘plus signs’ mean data has been collected.

- S2 Table: Although information on how DENV immune status of the participants was determined at the time of enrolment is described in the main text, this information should also be mentioned in the table.

**Response**: We agree with reviewer 1#, clarifying at this patient data was at the time of enrolment is important. The Table legend for S2 table now states: “***S2 Table****: Demographic characteristics of the reporting study population at enrolment. Includes suspected dengue case reports who reported in Indonesia (N:200), Vietnam (N:1,217) and the Philippines (N: 28,326).“*

2.b. Data analysis:
- Adding a figure describing the diagnostic algorithm (or rationale) used in the study would greatly benefit the manuscript.

**Response**: We agree with reviewer 1# and have now included an additional supplementary file that explains (with a flow diagram) how the DENV immune status of the reporting population was determined used laboratory methods. (See S1 File).

***Summary comments***

The goal of this manuscript was to determine and validate a framework to properly differentiate samples from primary and post-primary Dengue infections based on different combinations of points of care tests. The authors provided a well-conducted study with interesting results, however, the main limitations seem to be the choice of the standard serologic test and the lack of information regarding cross-reactivity with other flavivirus (e.g.; Zika). These two factors would be crucial to validate the authors' data and the proposed diagnostic framework. Further comments are presented in the previous sections of this review.

**Response**: We thank review 1# for their useful comments and suggestions, we hope our responses to the above specific comments answer their queries.

**Reviewer 2#**

Well written and interesting article on using combinations of tests to diagnose primary, secondary (+), historic or uninfected patients with suspected dengue. I have a few questions that I think would improve the manuscript if the authors could address them (immediately below) and then some minor points for their consideration.

It wasn’t clear why optimising a statistical measure (Youden’s J index) is the best way to select thresholds, especially if those thresholds are going to lead to decisions being made on clinical care. A more thoughtful determination of the relative importance of sensitivity and specificity is called for.

**Response**: Reviewer 2# raises an important point concerning the choice of statistical measure to determine thresholds. We used the Youden’s J index to calculate RDT +/- thresholds according to associated laboratory metrics which balances both sensitivity and specificity to minimise misclassification. Indeed, in clinical scenarios, it is often more important to prioritise sensitivity over specificity. I.e. it is often more important to capture everyone who could have the disease opposed to fewer individuals who have a higher chance of having the disease. In our study however, we were concerned with estimating laboratory test thresholds that would likely give rise to either a corresponding RDT negative or positive result. In our framework, being RDT+/- doesn’t necessary mean individuals would be classified as not having the disease, it would however impact their immune status classification. For instance, if we opted to use a statistical measure approach to determine thresholds that prioritised sensitivity, individuals with lower IgG ELISA titres would be classified as RDT IgG+, in turn, it would result in more primary DENV cases being categorised as RDT IgG+ (which in reality, they typically would not be). Our aim was to determine a laboratory threshold that gives the best possible representation of RDT positivity and negativity and minimise misclassification as much as possible. To make this clearer in the methods section, it now reads: *“…For each model, the optimal laboratory-derived metric cut off for RDT positivity was determined using Youden’s J index (sensitivity + specificity – 1) [38]. The threshold refers to the optimal estimated probability of being RDT positive according to sensitivity/specificity based on actual RDT outcomes. This approach was adopted to minimise the misclassification of RDT outcomes according to corresponding laboratory metrics….”* (See section: Methods, page 12, lines 372-377).

The authors have done logistic regressions of numerical test results to overall classification. I would have expected them to do two further things to demonstrate the validity of the logistic regressions: (1) to fit non-linear (on the logit scale) relationships for instance using splines to assure themselves and the reader that a linear relationship holds and/or (2) to present empirical estimates of the proportions to show that the logistic curve adequately characterises the data. This could be done through Hosmer-Lemeshow plots, or simply by aggregating all the points with IgM panbio below 10, from 10 to 20, 20 to 30 etc (say) and showing the empirical proportion and confidence interval is similar to that from the logistic regression.

**Response**: We agree with reviewer 2# that demonstrating that logistic regression modelling is appropriate for our data is necessary and we appreciate their suggestions. We have now run and included Hosmer–Lemeshow plots for each of the logistic regression models and justified them with good model fits. (See S1 File). In the methods, the HL tests are described: “…*To assess the validity of logistic regression modelling, Hosmer–Lemeshow tests were used to determine appropriate model fits (p-value>0.05).* (See section: Methods, page 12, lines 370-372). In the result section, the HL tests are referred to: *“..Models were assessed using Hosmer–Lemeshow tests which revealed no significant poor model fits (p-value>0.05)…”* (see section Results, page 14, lines: 434-435).

The combined framework leads to quite some uncertainty on the disease category (Table 3) for some combinations, such as disease day 1 and 2 with 1 positive RDT. If Table 3 is meant as a look up table, would there be no benefit to using the numeric values presented in Fig 2 to reduce the uncertainty? I thought it odd to coarsen the data at this early stage.

**Response**: Reviewer 2# highlights the high level of immune status uncertainty for some combinations of estimated RDT outcomes at the early stage of disease shown in Table 3, which indeed poses a challenge. For instance, among those presenting with only one estimated RDT result (out of the 3), cases often have an equal probability of being either primary or post-primary DENV. Reviewer 2# proposes utilising the data from figure 2 to reduce the uncertainty in table 3. In figure 2 however, the uncertainty is obtained from the probability of being RDT positive according to corresponding laboratory tests. (eg the probability of being IgG RDT positive according to IgG panbio units as measured by ELISA). Alternatively in Table 3, the uncertainly is associated the probability of being either a primary/post-primary/historical or negative dengue case according to different combinations of estimated RDT outcomes (by day of disease). As both Figure 2 and Table 3 represent different measures we are unable to propagate the uncertainty between them.

Moreover, we consider the uncertainly in table 3 to be of high importance. Many RDT commercial kits for example state an IgG- result is suggestive of primary DENV infection while an IgG positive result is indicative of a secondary infection. In our results, patients with estimated IgG- RDT result, particularly at the early stage of disease (days 1 & 2 of disease), could be either a primary or a post-primary case. This is likely due to the fact at the very early stage of disease, despite secondary cases having pre-circulating IgG in their blood from a prior DENV infection, the level of this IgG may not be high enough yet to elicit an IgG RDT+ result. This uncertainty is important to highlight as many health care workers may assume reporting cases with IgG RDT negative results are simply primary cases when they may be post-primary cases. In the discussion section we now highlight this: *“…We found that IgG RDT positivity was almost always confirmatory of a post-primary DENV infection. In contrast, an IgG RDT negative result on fever days 1 and 2 were suggestive of both primary and post-primary infections while at fever 3 to 5 were confirmatory of a primary infection. This infers simply classifying reporting primary and post-primary DENV cases according to IgG RDT negative and positive results, respectively, would lead to immune status misclassification…”* (See section Discussion, page 20, lines: 549-554).

Minor points:

182: Presumably the cases ‘sought’ care rather than ‘sort’ed care.

**Response**: changed

244: Awkward phraseology. “For instance, patients with a post-primary dengue infection could be given greater priority for monitoring and IHT than patients with primary dengue.” Would be better

**Response**: changed

255: Careful: elsewhere this is given as ‘days of disease’ rather than of infection.

**Response**: changed 'infection’ to ‘disease’

607: If ethics permits it, it would be preferable to make the data available online without needing the intercession of the corresponding author.

**Response:** We now clarify that “All relevant data are included within the manuscript and supporting information files.”

Figure 1: The whiskers on the bars are not defined. I assume they are 95%Cis. If so, several of the whiskers extend beyond the legal range (all six panels in panel B and both the panels in panel C [by the way, shouldn’t the panels be labelled a to p rather than a to c?]). Furthermore, some are seemingly deterministic (four bars in three of the panels in panels B and C). If the sample sizes are too small for the normal approximation to be valid then the authors should use a different way to construct confidence intervals.

**Response:** we agree with these suggestions for Figure 1. Panels have been relabelled A-P for clarity. Regarding the error bars, which originally represented 95%CIs based on normal distributions, we agree are not appropriate for these data. Given the small sample sizes here (most proportions have <30), it is not appropriate to generate confidence intervals based on normal distributions. Instead for figure 1, we calculated 2-sided confidence intervals based on t-distributions. Given how t-distributions gives rise to more conservative estimates (wider intervals), we opted to generate 2-sided 90% confidence intervals to help ensure the level of uncertainly remained in the legal range. This is now made clearer in the figure 1 legend (See Figure 1).

**Reviewer 3#**

***Methods***

-(261) Is there an EC approval from Vietnam?

**Response**: There is EC approval from Vietnam, we have made this clearer in the ethics section of methods: *“… This study was approved by the ethical review boards of the London School of Hygiene and Tropical Medicine (Ref: 17853), the Research Institute for Tropical Medicine (Ref: 2017-014), Nagasaki University/The National Institute of Hygiene and Epidemiology, Vietnam (Ref: VN01057) and Eijkman Institute for Molecular Biology (Ref: 136/2019).”*

-(263) How many verbals consent and assent were obtained (from 3 sites)?

**Response:** Unfortunately, we only had access to the number of participants enrolled into the separate studies, not the number of people/children who agreed to participate. We cannot confirm all those agreed to participate, were all subsequently enrolled at the clinics.

-(298) N: 29,634 (28326+1217+200=29743)

**Response**: This mistake has been corrected

-(310) NS1 RDT from Vietnam and Indonesia are different company?

**Response**: Indeed, different commercial kits were used in this study which we acknowledge is a limitation for this study. In the discussion we state: *“…In our study, we revealed individual laboratory metrics (PCR, IgM, IgG) were good predictors of corresponding NS1 IgM and IgG RDT outcomes. Despite this, we did observe some discordance between RDT results and laboratory metrics. For instance, several individuals with low antibody ELISA values still produced IgM/G RDT positive results. This might be due to the contrasting commercial brands used for the ELISAs and RDTs that rely on different epitopes present on DENV antigen that have contrasting immunogenicities*…” (see section: discussion, page 20, lines 568-573).

-(313,317) SD Biosensor, Abbott?

**Response**: This was a mistake, it now states just SD Biosensor.

-(323) How long IgM, IgG can be prolonged after infection?
-(333) Fever day 2 (PCR-, NS1-, IgM+, IgG+) = active post-primary or recent dengue infection?

**Response**: Reviewer 3# raises an important point regarding the antibody kinetics and classification of DENV immune status according to our algorithm. IgM is short-lived antibody which is a marker of current or recent exposure while IgG is a long-lived antibody that represents historical exposure to DENV. In our previously published article, we discussed the possibility of reporting cases presenting to a health facility, that were only IgM+, representing either a current or a recent DENV infections. However, given IgM offers temporary immunity to all serotypes following infection, those with recent exposure to DENV would unlikely report with dengue like-symptoms for a past infection. Therefore, we concluded if someone reported with suspected dengue symptoms, and had only elevated IgM, it is likely they have just surpassed the viremic stage of infection but they are still experiencing it. This is explained in our previously published article in the discussion section: *“…we classified those with active dengue infections as being either IgM or PCR positive given both rise and fall respectively during an active dengue infection [4, 5]. As dengue IgM persists for months following infection [4, 29], it could be argued our algorithm categorises recent dengue infections as active infections. However, elicited IgM provides temporary immunity to other serotypes and the study population included those seeking healthcare, so we considered it unlikely for individuals to seek treatment with dengue-like symptoms for a past infection…”* [1].

***Results***

-(S2 Table) Why Indonesia showed equal DENV1-4

**Response:** The Indonesian data used in this study was designed to allow a full comparison of laboratory and RDT tests, and did not reflect randomly collected patient samples, but rather selected patients for comparison purposes (as in our recent RDT assay comparison paper, [5]). We now make this clearer in our methods section: “… *In Indonesia, serum samples were collected from suspected dengue patients that reported across regions of Indonesia between July 2014 and July 2019 originally obtained for a previous study [37]*…” (See section Methods, page 10-11, lines 315-3317).

-S3 Table result came from which country?

**Response**: The data presented in table 3 refers to the estimated RDT results among patients from the Philippines where we didn’t have actual RDT data. This is made clearer in the table 3 legend: *“…Table 3: The probability of being primary, post-primary or historical for DENV according to every outcome combination of NS1, IgM and IgG RDTs stratified by disease day. RDT results estimated among patients from across the Philippines (N: 28,326*)…” (See Table 3)

-(433) Indonesia and Vietnam results were similar or different?

**Response**: As the Vietnam and Indonesia had separate data linked to individuals (See S1 table), we were unable to make a comparison between them. Our aim here was to estimate laboratory test (PCR/ELISA) thresholds that would likely give rise to corresponding RDT positive and negative outcomes. As Vietnam data had serotype-specific PCR and NS1 RDT data – we used this dataset to estimate the PCR (Ct threshold) that would give rise to a NS1 RDT positive result using logistic regression. Moreover, as there is a lag between viremia and NS1 secretion in hosts during an infection, we opted to stratify this analysis by disease day. Therefore, for each specific day of disease, we can estimate whether individuals likely have NS1 RDT +/- status according to their Ct value. For Indonesia, which was a smaller dataset, we had IgG/IgM ELISA and RDT data. We used this dataset to estimate IgG/IgM ELISA titres that correspond to IgG/IgM RDT positive/negative results, respectively.

-(445) Table 2 is the Vietnam result?

**Response**: Reviewer 3# identified a mistake in our results section where we mistakenly referenced the wrong table. It should have referenced table 1 at that point in the text. This mistake has been corrected.

-(460) Different NS1 RDTs (from Vietnam) showed similar agreement?

**Response:** The combined agreement in table 2 was only possible using the Indonesian dataset as this data included all NS1 IgM and IgG results (which were not all in the Vietnam dataset). We have now made this clearer in the text: *“…After generating DENV PCR and ELISA test thresholds that we estimated gave rise to NS1 and IgM/G RDT positive results, respectively, we explored how well our laboratory thresholds could estimate all 3 RDTs combined. Among the Indonesian sample population (N:200) who were tested using all 3 RDTs, we investigated the combined estimated RDT outcome agreement with the actual combined DENV RDT results (Table 2)…”* (See section Results, page 16, lines 493-497).

-(470) Why didn’t compare with the actual RDT result?

**Response**: Reviewer 3# queries why we didn’t compare our estimated Philippine RDT outcomes to actual RDT outcomes. This is due to fact in our larger Philippine dataset, we only have laboratory data (PCR, IgM and IgG ELISA data). Thus we utilised the Indonesian and Vietnam dataset to explore the translatability between RDTs and laboratory tests, using these results, we subsequently estimates RDT results among the Philippine samples based on their laboratory test values. This is now made clearer in the results section: *“… According to the optimal dengue laboratory metric thresholds, we estimated the NS1, IgM and IgG RDT positive and negative status of study population in the Philippines which lacked RDT data (N: 28,326)…”* (see section: results, page 17, lines 512-514).

-(471) Is there any change of being negative in Table 3

**Response:** In table 3, it shows the probability of being primary/post-primary/historical for DENV according to different combinations of estimated RDT+ results. In our findings, anyone with at least one RDT (included in the table) were either primary, post-primary or historical for DENV according to laboratory data. No one with an estimated RDT positive result were negative for DENV.

***Conclusions***

-(495) Diagnosis of acute dengue infection from suspected dengue patients need confirmed lab test (PCR+) before combination of RDTs results can give clear predictions of immune status.

**Response**: Agreed. All suspected cases in this dataset received laboratory testing (PCR, IgM and IgG) to determine whether they were active with DENV prior to immune status classification (see S1 file).

-(520,528) Please also compare with both NS1 RDTs companies.

**Response:** In our study, we were unable to compare how well both commercial NS1 RDTs correspond to viremia (CT). As these represent indirect measures (NS1 RDTs detect secreted NS1 and CT value correspond to the amount of nucleic acid in serum), we had to stratify logistic regression models by day of disease to account for the lag between viremia and secreted NS1 during an infection. This was possible using the Vietnam dataset yet was not feasible with the Indonesian dataset, so we couldn’t compare accurately both companies. Despite this, the performance of these NS1 RDT has been conducted previously and found these brands to have adequate sensitivity and specificity for capturing PCR-confirmed DENV infections [5].

-(552) How many severe dengue in primary and post-primary?

**Response**: In this study not all those included had symptom data linked to laboratory data (S1 Table). In our previous paper which we reference in this manuscript, we do give the numbers of primary and post-primary cases with severe disease and show post-primary, opposed to primary, DENV is a risk factor for severe disease. [1,6]

***Summary comments***

-The paper is interesting but difficult to follow so please include study flow chart.

**Response**: After addressing the reviewers comments, we have made substantial changes to our manuscript which we believe makes the article easier for readers to follow. We refer Reviewer 3# to the updated S1 table which provides more explanation as to what data was available from contrasting datasets. The lack of RDT data among the large Philippine dataset is the reason we had to first determine laboratory test threshold (ELISA and PCR) that could be used to estimate RDT outcomes in table 3 – our framework to categorise immune status according to combined RDT results.

-What are the reasons for multiple sites without comparison?

**Response:** As data from different sites included contrasting data (not all datasets had RDT data) we were unfortunately unable to make a comparison between them. We did however compare the epidemiological characteristics and immune status classifications between sites (based on lab methods NOT RDTs) in S2 Table.

**References**

1.	Biggs JR, Sy AK, Brady OJ, Kucharski AJ, Funk S, Reyes MAJ, et al. A serological framework to investigate acute primary and post-primary dengue cases reporting across the Philippines. BMC Med. 2020; 18(1):364.

2.	Blacksell SD, Jarman RG, Gibbons R V., Tanganuchitcharnchai A, Mammen MP, Nisalak A, et al. Comparison of seven commercial antigen and antibody enzyme-linked immunosorbent assays for detection of acute dengue infection. Clin Vaccine Immunol. 2012; 19(5):804–10. PMID: 22441389

3.	World Health Organisation (WHO). Evaluation of commercially available anti-dengue virus immunoglobulin M tests. Avaliable at: https://www.who.int/tdr/publications/documents/diagnostics-evaluation-3.pdf. (Accessed 01/03/22) [Internet]. 2009. 26–28 p.

4.	Biggs JR, Sy AK, Brady OJ, Kucharski AJ, Funk S, Tu Y-H, et al. Serological Evidence of Widespread Zika Transmission across the Philippines. Viruses. 2021; 13(8):1441. PMID: 34452307

5.	Santoso MS, Yohan B, Denis D, Hayati RF, Haryanto S, Trianty L, et al. Diagnostic accuracy of 5 different brands of dengue virus non-structural protein 1 (NS1) antigen rapid diagnostic tests (RDT) in Indonesia. Diagn Microbiol Infect Dis. 2020; 98(2):115116. PMID: 32679344

6.	Biggs JR, Sy AK, Sherratt K, Brady OJ, Kucharski AJ, Funk S, et al. Estimating the annual dengue force of infection from the age of reporting primary infections across urban centres in endemic countries. BMC Med. 2021; 19(1):217.
